# Parallel Optimization of 3D Cardiac Electrophysiological Model Using GPU

**DOI:** 10.1155/2015/862735

**Published:** 2015-10-25

**Authors:** Yong Xia, Kuanquan Wang, Henggui Zhang

**Affiliations:** ^1^School of Computer Science and Technology, Harbin Institute of Technology, Harbin 150001, China; ^2^Biological Physics Group, School of Physics & Astronomy, University of Manchester, Manchester M13 9PL, UK

## Abstract

Large-scale 3D virtual heart model simulations are highly demanding in computational resources. This imposes a big challenge to the traditional computation resources based on CPU environment, which already cannot meet the requirement of the whole computation demands or are not easily available due to expensive costs. GPU as a parallel computing environment therefore provides an alternative to solve the large-scale computational problems of whole heart modeling. In this study, using a 3D sheep atrial model as a test bed, we developed a GPU-based simulation algorithm to simulate the conduction of electrical excitation waves in the 3D atria. In the GPU algorithm, a multicellular tissue model was split into two components: one is the single cell model (ordinary differential equation) and the other is the diffusion term of the monodomain model (partial differential equation). Such a decoupling enabled realization of the GPU parallel algorithm. Furthermore, several optimization strategies were proposed based on the features of the virtual heart model, which enabled a 200-fold speedup as compared to a CPU implementation. In conclusion, an optimized GPU algorithm has been developed that provides an economic and powerful platform for 3D whole heart simulations.

## 1. Introduction

With rapid advances in imaging modalities, computer models of the whole heart become more sophisticated with detailed anatomical structures with high spatial resolution, which are integrated with detailed cardiac electrophysiology. With a spatial resolution higher than 150 micrometers which is equivalent to the length of a cardiac myocyte, a 3D heart model can easily have discrete elements more than tens of millions or even billions. Given the fact that for each element a few dozens of state variables are required to describe the electrical activity, ion channel kinetics, and ion concentration homeostasis, simulation of cardiac tissue with real heart geometry and detailed electrophysiology is large-scale, imposing a big challenge for computation power and resources.

For large-scale cardiac modeling, systems of high-performance computing (HPC) with tens to hundreds of CPUs have been used. In their study, Niederer et al. [1] implemented an algorithm of HPC with 16384 CPUs to simulate human cardiac electrophysiology. A simulation of 1000 millisecond cardiac electrical activity was performed within 5 minutes, a time scale closer to practical clinical applications. However, uses of HPC, either OpenMP (Open Multi-Processing, http://openmp.org/) [2] or MPI (Message Passing Interface, http://www.mpi-forum.org/) [3–5] systems, can have a very obvious speedup in simulations after overcoming the difficulties of multithreads programming, but they are still cost-ineffective due to their high price and operation complexity, each of which hinders their practical applications.

GPU, the widely available Graphical Processing Units (GPUs), can offer a cheap, convenient alternative to large numbers of CPUs, providing a cost-effective parallel computing technology in a standalone desktop PC environment. Over the last decade, especially after the launch of Compute Unified Device Architecture (CUDA) by Nvidia cooperation in 2007, GPU has been widely used for general computing including large-scale cardiac simulations [6–11]. It has been shown that tens of speedup factors as compared to the CPU can be achieved in monodomain and bidomain models of cardiac tissue with biophysically detailed mathematical equations of cardiac electrical activities [7, 8]. Whilst implementation of single precision float showed better speedup performance than that of double precision float, it yielded a loss of simulation accuracy [9]. In their study, an about 70-fold speedup was obtained by Vigueras et al. [10] and Mena and Rodriguez [11], which was close to that obtained by us in a previous study [12]. A higher speedup performance was achieved by using multiple GPUs [13, 14]. In addition, CUDA showed better speedup performance in cardiac simulations than other programming languages, such as OpenCL. Though OpenCL is a more portable way of programming for scientific computing problem, it is not as efficient as CUDA when running on Nvidia GPUs for cardiac simulations [15, 16].

Because of differences between CPU and GPU architectures, it is not trivial to port CPU programs of cardiac models to GPU directly as some special considerations are needed. Although the platforms developed by Lionetti et al. [17] and Amorim et al. [18] provided a tool for automatically porting cardiac simulation codes from CPU to GPU for users without detailed knowledge of GPU, such automatically generated CPU codes are not optimized and have low efficacy.

An optimized GPU performance of cardiac simulation codes can be achieved by special considerations of data structure and algorithms of cardiac models. In this study, we presented an optimized GPU code of 3D model of the sheep atria [19] by using a recently launched GPU K40 system (tone the Tesla series). The optimized GPU algorithm achieved up to 200-fold speedup as compared to the CPU counterpart. In the sections below, we present some numerical strategies and the GPU optimization skills in detail.

## 2. Numerical Methods for the Sheep Atria Model

### 2.1. 3D Model of the Sheep Atria

Sheep are often used as animal models for experimental studies into the underlying mechanism of cardiac arrhythmias. Recently, we have developed a family of mathematical models for the electrical action potentials of various sheep atrial cell types [19]. The developed cell models were then incorporated into a three-dimensional anatomical model of the sheep atria, which was recently reconstructed and segmented based on anatomical features within different regions. This created a novel biophysically detailed computational model of the three-dimensional sheep atria [19].

Although bidomain model was the most general and accurate description for cardiac electrical activity, the complexity of computation caused much trouble for application. In fact, the bidomain model could be reduced to monodomain model based on some assumptions [20].

In this paper, the action potential propagation throughout the 3D sheep atria tissue was simulated using the monodomain reaction-diffusion equation, and the atria model can be described by

(1) dVdt=∇·D∇V−Iion+IstimCm,

(2) Iion=INa+ICa+INaK+IClCa+IKr+IKs+Ito+IKurd+INaCa+IpCa+IbNa+IbCa+IK1,

where *V* is the transmembrane voltage, *I*_ion_ is the total ionic current, *I*_stim_ is the stimulate current, *C*_*m*_ is the transmembrane capacitance, and *D* is the diffusion tensor.

### 2.2. Numerical Solvers

The model described in (1) is a set of nonlinear coupled system including ODEs and a PDE. To solve the equation, the operator splitting technique in [21] was used for discretization. In order to implement GPU parallelization, we split the model into two parts: single cell model with ODEs for describing the cellular electrical activity of cardiac cells and the PDE model for describing the intercellular electronic interactions between cells, which was defined as

(3) dVdt=−Iion+IstimCm,

(4) dVdt=∇·D∇V.

For each time step, the ODE solver and PDE solver were executed in turn, and two kernel functions were used for the ODE solver and PDE solver separately. A finite difference discretization was used to discretize space. And a forward Euler method [22] with a time step 0.005 ms was implemented to solve the equations, which gave accurate results as compared to the solutions reported in our previous study [19].

#### 2.2.1. ODE Solver

First of all, equilibrium potentials for Na^+^, K^+^, Cl^−^, and Ca^2+^ ionic channels were updated based on the current transmembrane voltage and their initialized parameters. Then, various ionic currents and the total current were updated. Furthermore, the transmembrane voltages were updated based on the forward Euler method for (3). After transmembrane voltage update, the ionic concentrations were also updated by the forward Euler method. At last, various gate control variables are updated based on the following method.

For a gate control variable *x*, the governing equation was defined as

(5) $$\begin{array}{c} \frac{dx}{dt}=\alpha_{x}\left(\;V_{m}\;\right)\left(\;1-x\;\right)-\beta_{x}\left(\;V_{m}\;\right)x. \end{array}$$

According to the Rush-Larsen method [23], the solution of *x* was obtained by

(6) $$\begin{array}{c} x\left(\;t+\Delta t\;\right)=x_{\infty }-\left(\;x_{\infty }-x\left(\;t\;\right)\;\right)\cdot e^{-\Delta t/\tau_{x}}, \end{array}$$

where

(7) x∞=αxVmαxVm+βxVm,τx=1αxVm+βxVm.

Because the computation of ODE solver was only for a single cell and the coupling between cells could be ignored, the ODE solver was very ideal for parallelization.

#### 2.2.2. PDE Solver

In order to match the actual electrophysiological conduction of excitation waves in the sheep atria, a 3D anisotropic diffusion tensor was used. The produced activation time sequence within the model and the measured conduction velocities of the atria matched the experimental data as well as those reported in our previous study [19], validating the performances of the model. For the 3D anisotropic tissue, the diffusion tensor *D* was defined as

(8) $$\begin{array}{c} D=\left(\begin{array}{ccc} D_{xx} & D_{xy} & D_{xz}\\ D_{yx} & D_{yy} & D_{yz}\\ D_{zx} & D_{zy} & D_{zz} \end{array}\right). \end{array}$$

Then, (4) was transformed to

(9) $$\begin{array}{c} \frac{\partial V}{\partial t}=\underset{i,j=x,y,z}{\sum }\left[\;\frac{\partial }{\partial i}\left(\;D_{ij}\frac{\partial V}{\partial j}\;\right)\;\right]. \end{array}$$

The forward Euler method was used for the discretization of (9) in the time domain. The tissue space was discretized by a finite difference method with a space step of 0.33 mm, the same as in our previous study [19]. The fibre orientation vectors were averaged to provide local directions of fibres in 0.9 mm^3^ voxels. A diffusion ratio of 6 : 1 (parallel : perpendicular to fibre direction) was chosen for the best matching of anisotropic conduction velocities to electrophysiological experimental data, with which *D*_||_ = 0.3 mm^2^/ms, *D*_⊥1_ = *D*_⊥2_ = 0.05 mm^2^/ms were used, respectively.

The computation of PDE solver was based on a 3-dimensional array. It might cause some troubles for data access efficiency on GPU platform, therefore increasing the computing time without special considerations of GPU optimization. Below, we described some optimization strategies of the GPU algorithm.

## 3. Programming on the GPU

### 3.1. GPU Architecture

To optimize the GPU code, we first need to know GPU architectures. A modern GPU is a highly data-parallel processor, containing several or tens of hundreds of processors running in a Single Instruction Multiple Data (SIMD) model, which reduces computing time by letting the same operation being carried out simultaneously on each of the multiple threads with very little memory overhead. Though this allows near-simultaneous calculation of many independent floating point operations, it does suggest that the GPU architecture is suitable only for independent and data-parallel computations.

The CUDA architecture enables a GPU as a data-parallel computing device. A CUDA program consists of two parts including the CPU part (host) and the GPU part (device). The host runs nonparallel or simple task by allocating memory for host and device and transferring data between host and device. Kernel function is executed in device, which is decomposed into blocks that are allocated to stream multiprocessors. Furthermore, there are multiple threads within one block which can be set by developer. All threads within one block will get the same computation resources, and the communication between threads is based on share memory in multiprocessor or global memory. One thread will be mapped to one processor. In particular, all the threads within a block are grouped into warps and a warp generally includes 32 threads, which can run concurrently in the best case.

### 3.2. Programming Procedures for GPU

For GPU programming, CUDA C++ was used. As being described in Section 2, models for the electrical activity in cardiac tissue were decomposed into two parts. One was for the single cell model which was suitable for GPU parallelization, and the other was for the interaction between cells, which was unsuitable for GPU parallelization. For the 3D atrial model, each mesh node represented a single cell. Simulation flow was divided into three phases: preprocess, iteration and data refreshing, and postdata process. In the preprocess, model initialization, including mesh building, definition of mesh data, and mapping of fiber orientation, was conducted. This process was implemented on the CPU. The process of iteration and data refreshing includes computations of cell excitation, state-variable updating from ODE, and PDE solvers. This part of computation was executed recursively, forming a vital part for the GPU speedup. Finally, postprocess was used for data storage and analysis, which runs on the CPU.

Details of each of the procedures were described below.

Preprocess (run on host) is as follows.

1. Build mesh including data structure for single cell and diffusion tensors between cell neighbors and allocate memory for mesh.
2. Read fiber orientation and cell categories of each cell node.
3. Map fiber orientation and cell categories to meshes.
4. Compute diffusion tensors between cell neighbors based on fiber orientation.
5. Allocate memory for device.
6. Copy data from host to device.

Data iteration and refreshing (run on device) is as follows.

One finds the following.

(7) Initialization of state-variable data in device.
(8) Cell stimulation: all cells of sinoatrial node were stimulated by setting their action potentials to +20 mV at the beginning.
(9) Iteration for a certain duration.
  (a) ODE solver, state-variable updating, and important data saving.
  (b) PDE solver, state-variable updating, and important data saving.
  (c) Save action potentials of all cells for a next time step.

Postprocess (run on host) is as follows.

(10) Copy data from device to CPU.
(11) Free device memory.
(12) Analyze the data.
(13) Save necessary data to files.
(14) Free host memory.

## 4. GPU Optimization Strategies

For cardiac tissue simulations, at each iteration step, intensive computations are required for tens of state-variable loading, updating, and storage. Therefore, data structure for storage is a key factor for the speedup of simulations. In addition, a reasonable allocation of the threads is also an important factor. In this section, we present some strategies to optimize the GPU parallelization by considering the memory usage and thread arrangement. Although other optimization strategies improve the GPU performance, their role was less significant compared to the memory usage and thread arrangement.

### 4.1. Optimization of Memory Access

At each iteration, though the computational complexity for each individual cell was not high, the total computational cost was high due to the large number of cells. In addition, the total number of model parameters was over a hundred, and many of them were voltage-dependent and required to be updated and saved at each iteration. It was obvious that the efficiency of data storage, reading, and writing was a key problem for the speedup of the GPU code.

There are a variety of storage media in the GPU, mainly including register, shared memory, multistage cache, local memory, and global memory. Local memory is a part of global memory in fact, so the technique characteristic of these two memory types is the same. Among all memory media, register is the fastest, but the number of register is very limited, up to 64 K in a stream multiprocessor. Register is mainly used for the allocation of some local variables in kernel functions or device functions. The speed of shared memory is also very quick, but the memory size is also very small, about dozens of KB in a stream multiprocessor. Shared memory is mainly used for communication of threads within a block. Global memory is the slowest, but the storage capacity is large. There is over 1 GB memory size for mainstream GPU card. In particular, memory size of 12 GB can be found in some recently launched GPU cards, such as K40, a device from Tesla series.

For millions, even tens of millions of myocardial cells, model parameters demand storage space of several GB, even more. Such a large memory demand can only be satisfied by using global memory. In order to improve the efficiency of reading and writing for global memory, we present three optimization ways: one is to cut the number of variables that need to be stored; the second is to use an appropriate design of data storage structure; and the third is the appropriate use of registers, shared memory, and cache to improve the speed of memory reading.

#### 4.1.1. Variables Decrease

The time for the reading or writing of global memory is more than 400 machine cycles. For the sheep atria model, there are more than 100 variables and parameters used for each iteration. If all variables were saved in global memory, the efficiency of variable access was very low. In fact, some variables were not necessary to be saved in global memory, which could be updated based on other variables, not itself. As the bottleneck of GPU speedup was the memory access, we analysed the model aiming to reduce memory access. It was found that about 60 variables and parameters need to be arranged in global memory, among them about 40 variables required to be read and written; about 20 variables only required to be read. If there were 10 M mesh nodes considered, the required memory size was 10 M*∗*60*∗*8 = 4.8 GB. If time step was set to be 0.001 ms, there was about 1 million iterations for 1 s simulation. It was obvious that the memory access required to be considered more thoroughly.

#### 4.1.2. Data Structure of Storage

**Figure 1:**
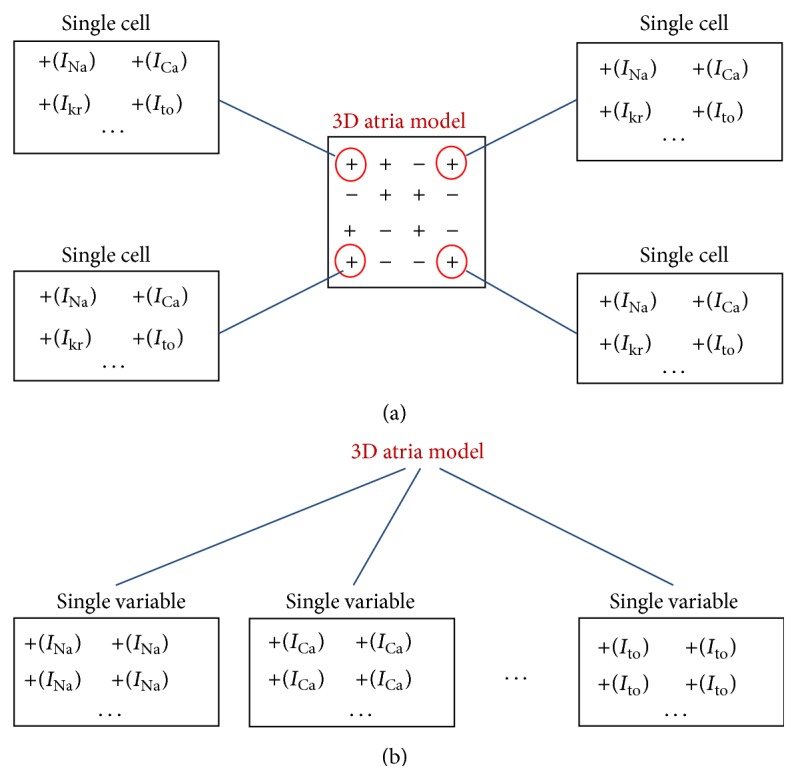
Data structure comparison between CPU and GPU. (a) CPU with heterogeneous storage of model parameters; (b) GPU with homogeneous storage of model parameters reducing memory access time for each iteration of simulations.

In the program of CPU version, we defined a class for each cell mesh, all parameters of cell, including action potential, current, and ionic concentration, were declared as member variables within the class, which was very convenient and clear for programming. But for GPU version, the data structure of cell class was very low in efficiency. The reason is that memory access in single thread programming is very different from that in multithread multiprogramming. In general, for the present mainstream GPU, one operation of memory reading will get continuous 128 bytes. Based on this fact, we constructed a continuous memory access for variables and parameters in multithread as shown in Figure 1.

Figure 1 illustrated the data structure for CPU (Figure 1(a)) and GPU (Figure 1(b)). In the GPU implementation, parameters of cells in the 3D spatial geometry were split into multiple arrays of 3D parameters with one array containing one homogeneous parameter, allowing a continuous storage of the parameter, which improved the efficiency of data access on the GPU.

#### 4.1.3. Sparse of Data

Furthermore, a linear and compact indexing of mesh nodes helped to increase GPU implementation efficacy. In the 3D atria, there were many mesh nodes representing atrial cavity or blood, which could be ignored directly by reindexing the 3D mesh into a 1D linear and compact cell storage array. In the 3D model, a variety of different cell types were used to represent the intrinsically electrical heterogeneity of the atria including left atrium, right atrium, pectinate muscles, Bachmann's bundle, and pulmonary veins and the empty cavity. During kernel function of ODE solver or PDE solver, cell type was required to be launched first. If the node was for an empty cell, then return without performing computing. This process might cause some problems for multithreads programming. First, the thread of empty cell was meaningless and wasted resources and time. Second, continuous 32 threads within a warp existed in the process of branching calculation of different cell types, which caused the threads within a warp not to be synchronized. Therefore, without use of a compact indexing reduced the computational parallelism. So, it was necessary to reindex the 3D mesh in a compact format by omitting the empty cells in order to enhance the GPU performances.

### 4.2. Thread Arrangement

Appropriate arrangement of threads can effectively improve the occupancy rate of the GPUs. In order to effectively use the register, each processor should have at least 192 active threads, which can reduce the effects of the time delay of writing after reading. In order to guarantee the GPU resources occupancy rate close to 100%, the number of threads run on a stream multiprocessor should be more than 192.

As each block is mapped to a stream multiprocessor, the number of blocks should be arranged not less than the number of stream multiprocessors. In particular, in order to avoid the waste of resource due to block blocked, the number of blocks should be as many as possible, such that the blocks with different states overlap on the timeline. The later experimental results also illustrated this point.

Due to the fat that the warp is the minimum unit for thread scheduling on the GPU, and the number of threads within a warp is 32, and in order to improve the utilization rate of resources, the number of threads within a block should be the multiple of 32. For more than one stream multiprocessor, we should reasonably arrange the number of blocks and that of threads within a block. In this study, we adopted various different thread configurations for performance test. Results were presented in the result section.

For the design of thread, the strategy of the ODE solver is different from that of the PDE solver. For ODE, only single cell needs to be considered for one thread. So, the number of valid cells is set as the number of threads and a simple 1-dimensional thread traverse is used. However for the PDE, it is more convenient if a 3-dimensional thread traverse structure is used because the computation of a cell is related to 26 adjacent cells. So, the number of all cells is set as the number of threads. Because all parameters of cell have been saved by compact storage as described in Section 4.1.2, a mapping table from 1 dimension to 3 dimensions needs to be constructed.

## 5. Experimental Results

We used a computer with CPU @ Intel Ivy Bridge E5-2620v2/2.1 GHz and a Nvidia GPU card K40 for testing the developed CUDA algorithm. The simulated data was visualized and compared to the results from our previous study [19] to validate the correctness of the GPU algorithm. Furthermore, various optimization strategies on GPU performance were evaluated.

### 5.1. Simulation Results

In GPU implementation of the 3D sheep atrial model, nine different cell types were defined and different initializations were done for different cell types as done in our previous study [19]. The model consisted of about 7.1 million mesh nodes (317 × 204 × 109). Because the atria have very thin walls, the number of valid cell nodes was actually only about 1 million. Therefore, the data was sparse and special strategy was needed for data access and storage for the best performance. Each node was mapped to a cell model according to the segmentation of the tissue [19]. Double precision for parameters was used for guaranteeing simulation accuracy. There were over a hundred parameters and state-variables for each node, 60 of which were saved in global memory on the GPU for frequent reading during simulations. There were about 40 state-variables requiring update and storage for each iteration mainly for ODE solver. We tested the GPU algorithm efficiency in a simulation duration of 600 ms with a time step of 0.005 ms. The action potential of each node was saved at a 5 ms interval (i.e., every one thousand time steps) for data analysis and visualization off-line.

**Figure 2:**
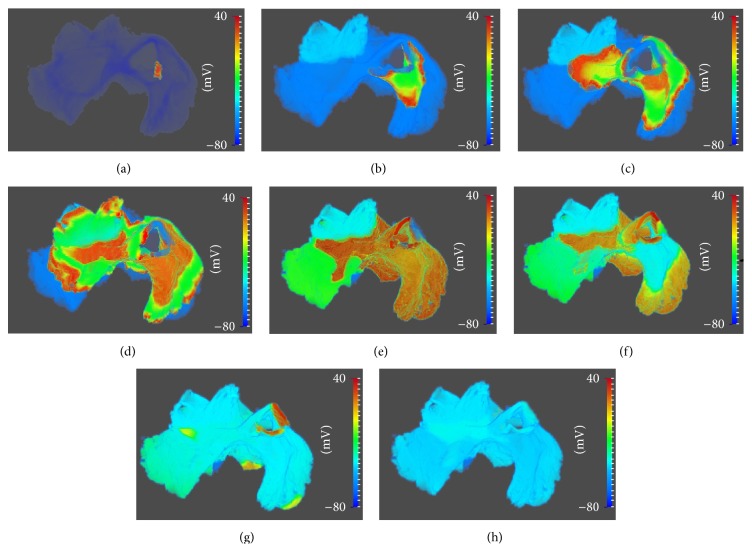
Snapshots of propagating action potentials across the whole 3D atria. (a) 0 ms; (b) 15 ms; (c) 30 ms; (d) 45 ms; (e) 90 ms; (f) 120 ms; (g) 150 ms; (h) 210 ms.

Figure 2 showed the simulated excitation wave propagation in the 3D sheep atria. The electrical wave was first initiated in the central sinoatrial node region. Then, it diffused to all tissue in the right and left atria. After 200 ms, the atria completed repolarization and returned to its resting state. The activation pattern of excitation waves is consistent with the sheep atria electrophysiology and matches the simulation data of Butters et al. [19] by using CPU algorithm. This illustrated a successful implementation of the GPU code for 3D sheep atrial model. Below, we investigate the effects of different optimization strategies on the GPU code performances.

### 5.2. Effects of Variant Optimization Strategies on GPU Performance

For large-scale cardiac tissue models (about dozens of millions of nodes, each with hundreds parameters/state-variables), optimizing the performance of memory access plays a key role for improving the whole performance. Meanwhile, as the arrangement of the threads affects the occupancy rate of resources, it also plays an important role in the improvement of GPU performance. To demonstrate this, we compared the performance of three different optimization methods, including

 GPU 1: a direct program transferred from CPU to GPU (GPU 1, continuous access for cell class);
 GPU 2: adaptation of the cell data structure (GPU 2, continuous access for variables);
 GPU 3: the overall optimization (GPU 3, variant strategies including variable decrease, data storage, and sparse of data).

**Figure 3:**
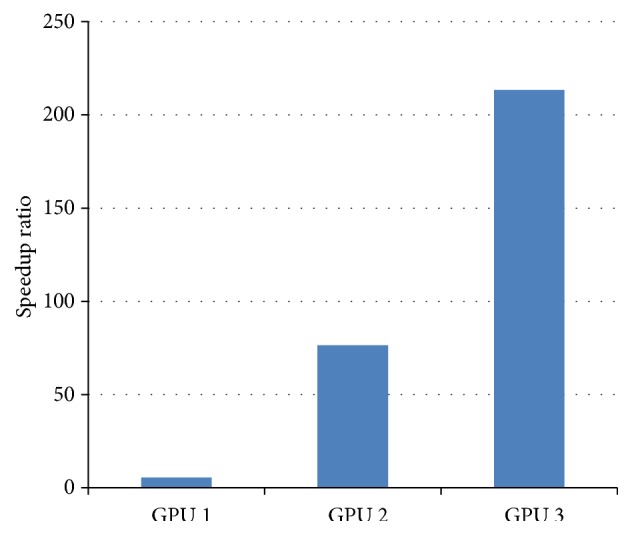
Performance of speedup for variant GPU optimization strategies.

To investigate the efficiency of the GPU s1, 2, and 3 implementation schemes, the execution time was measured for simulating 600 ms atrial electrical excitation waves by using the 3D atrial model and the number of threads was fixed to 256. The speedup of GPUs 1–3 schemes was calculated in relation to the execution time of CPU. Results were shown in Figure 3, and *x*-axis is variant optimization strategies on the GPU; *y*-axis represented speedup ratio of execution time between GPU and CPU. It was shown that a direct program transfer from CPU to GPU was not efficient due to the different architectures between the two. Considering GPU architectures characteristic for continuous access of variable as discussed in Section 4.1, the GPU performance was improved dramatically as shown in the case of GPU 2. By further optimization, by considering more strategies in Section 3.1, a more than 200-fold speedup was obtained, which reduced the execution time from 360000 seconds in CPU to 1687 seconds in the GPU 3 case. Furthermore, it was shown that the speedup from GPU 1 to GPU 2 was significant. Therefore, for our application, as a massive data required to be read and written at each time step, the efficiency of data access played a key role in the speedup of the algorithm.

### 5.3. Effects of Different Data Size on GPU Performance

**Figure 4:**
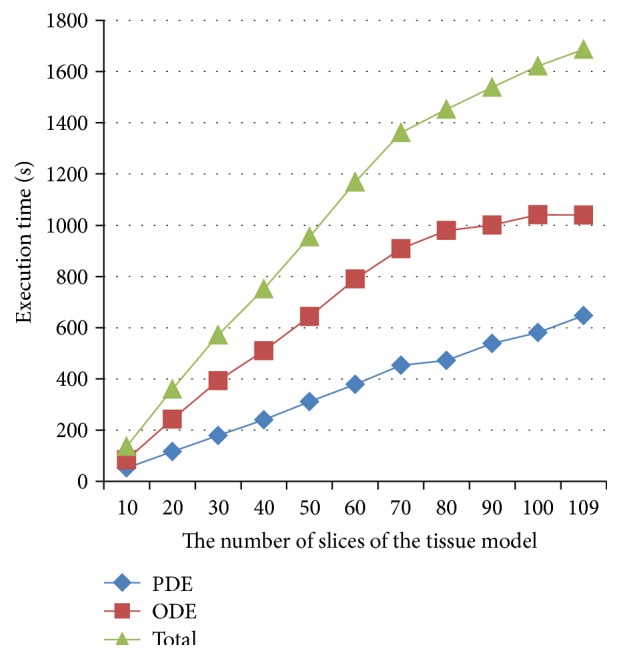
Effects of data size on the execution time of PDE, ODE solvers, and the total computing.

We also considered the effect of data volume on the GPU performance. In the whole 3D sheep atrial model, the anatomical structure consists of 109 slices in the *Z* axis. By varying the size of the geometry (i.e., the number of slices) in simulation, the execution time was measured for the PDE and ODE computations and the total of the two. Results were shown in Figure 4, and the *x*-axis represented the number of slices of 3D model and *y*-axis the execution time. It was shown that with the increase of data volume, the increase in the measured execution time flattens, indicating that the parallelization efficiency was better with an increased data volume. This was possibly due to the fact that when data size became greater, the occupancy of resource was increased, leading to an increased hit ratio of the cache.

### 5.4. Effects of Arrangement of Thread and Block on GPU Performance

**Figure 5:**
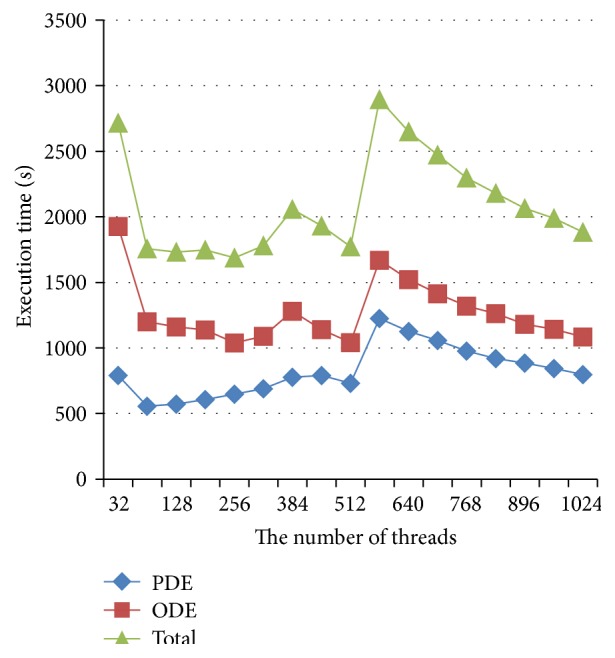
Effects of the numbers of threads on the execution time.

We investigated the effects of arrangement of thread and block on GPU performances. The results were shown in Figure 5, in which the execution time (*y*-axis) was shown against the number of threads (*x*-axis). It was shown that the number of threads (*x*-axis) had obvious effects on the execution time. In testing, the number of blocks used was adaptively changed with different thread arrangement as follows:

(10) $$\begin{array}{c} bn=INT\left(\;\frac{cn+tn-1}{tn}\;\right), \end{array}$$

where cn is the total number of cells in the model, tn is the number of threads in a block, bn is the number of blocks, and INT() is a function which evaluates a numeric expression and returns the integer portion of the expression.

With a bigger number of blocks, the waste of resource became more pronounced. And vice versa with a smaller size of block the waste of resource was reduced, but this led to a reduced efficiency of memory reading. As one reading operation gets 128 bytes data, if the block size is less than 128, the efficiency of data access cannot reach the best optimization. From Figure 5, it is shown that with 64 threads each block was the best for the PDE solver, and with 256 threads each block was the best for ODE solver. If the same threads were set for both the PDE solver and the ODE solver, then with 256 threads each block was the best for the whole simulation.

**Figure 6:**
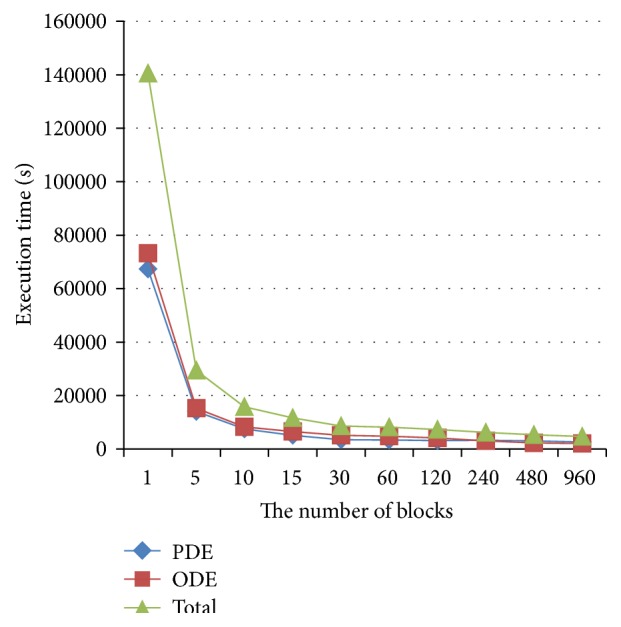
Effects of block number on the execution time of the GPU algorithm.

Effects of the number of processors on GPU performance were also investigated. Results were shown in Figure 6, in which the *x*-axis represented the number of blocks and *y*-axis the execution time. As it was not possible to directly control the active number of processors, the test was done indirectly by controlling the block configuration. In this test, the block size was fixed with a block consisting of 256 threads, and the number of blocks increased from one to 960. Our results suggested an exponential decrease of execution time with the increase of the block number.

Figure 6 showed a biphasic linear speedup of the GPU performance with the increase of block number. In the first phase, the block number was less than 15. In this case, due to equal allocation of blocks to stream multiprocessors, there was just one block running in each stream multiprocessor. The performance improvement was obvious due to the increased number of stream multiprocessors.

**Figure 7:**
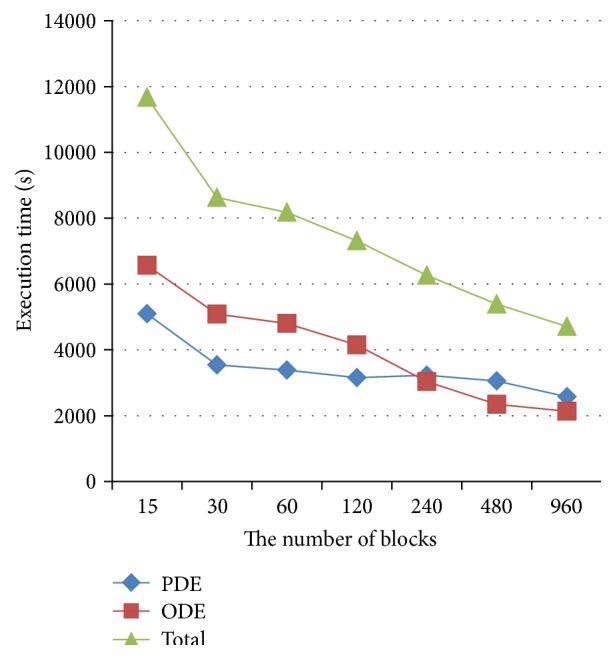
Effects of block number on the execution time for block number more than 15.

When the number of blocks was more than 15 and continued to increase, the number of processors remained the same, but the performance was still improved dramatically as shown in Figure 7. The reason was due to the fact that more thread allocation to a stream processor could be beneficiary for performance improvement as less threads in a block might cause resource waiting or even thread being blocked. For the Nvidia K40, the maximum of threads used could be up to 2048, but only 192 processors could be considered in one stream multiprocessor.

Therefore, one should make the number of threads in stream multiprocessor to attain the maximal performance of the algorithm by reasonable arrangement of configuration of thread and block. Reasonable programming of multithread is required to increase the number of active threads, which is proportional to speed performance. However, such speedup is not simply linearly related to the number of active threads. Besides, the efficiency of memory access is another important factor for speed performance, which even becomes the bottleneck of the technique for some applications with frequently memory access.

## 6. Conclusion

It is still a challenge to develop an efficient and economical parallel computing algorithm for cardiac simulation given the fact that the models are becoming more and more sophisticated with detailed electrophysiology and anatomical structures. In particular, for the aim of patient-specific personalized cardiac modeling, fast simulation speed as real- or near real-time simulations is expected. In this study, we developed an efficient GPU CUDA algorithm based on several proposed optimization strategies regarding the optimal use of data access, data structure, and balanced allocation of thread. With such optimization strategies, up to 200-fold speedup was achieved as compared to CPU implementation. This speedup is dramatically better than the previous reported speedup of about 70-fold [11, 12] by using a single GPU. Though an up to 400-fold GPU speedup was achieved in a previous study [13], that was using multiple GPUs. Effects of such optimization strategies on improving multiple GPU algorithm performance warrant further studies in soon future. In conclusion, we have developed optimization strategies to improve the performance of GPU algorithms, which provides a powerful and economical platform for large-scale cardiac modeling.
